# Comparative transcriptomics and bioinformatics analysis of genes related to photosynthesis in *Eucalyptus camaldulensis*

**DOI:** 10.7717/peerj.14351

**Published:** 2022-11-11

**Authors:** Ni Zhan, Liejian Huang, Zhen Wang, Yaojian Xie, Xiuhua Shang, Guo Liu, Zhihua Wu

**Affiliations:** 1 Research Institute of Fast-growing Trees, Chinese Academy of Forestry, Zhanjiang, Guangdong, China; 2 Langfang Normal University, Langfang, Hebei, China; 3 Research Institute of Tropical Forestry, Chinese Academy of Forestry, Guangzhou, Guangdong, China

**Keywords:** *Eucalyptus camaldulensis*, Transcriptional sequencing, Gene analysis, Photosynthesis

## Abstract

The timber species* Eucalyptus camaldulensis* is one of the most important in southern China. Therefore, it is essential to understand the photosynthetic pattern in eucalyptus leaves. In the present study, eighteen photosynthesis-related genes were analyzed using bioinformatics methods. The results indicated that there were ten differentially expressed *ribose-5-phosphate isomerase* genes (*RPI*), and six of them were up-regulated in the mature leaves compared to the young leaves, while others were down-regulated. The differential expression of four *rubisco methyltransferase* genes (*RBCMT*) were observed. Two of them were up-regulated, while two were down-regulated in mature leaves compared to young leaves. Furthermore, two *ribulose-phosphate-3-epimerase* genes (*RPE*) were up-regulated in the mature leaves compared to the young leaves. In contrast, two genes involved in *triosephosphate isomerase* (TIM) were down-regulated in mature leaves compared with young leaves. The current study provides basic information about the transcriptome of *E. camaldulensis* and lays a foundation for further research in developing and utilizing important photosynthetic genes.

## Introduction

Photosynthesis, as an important physiological activity of plants, changes its characteristics and can effectively reveal the internal physiological state of plants as well as regulate the ecological adaptation to their habitats (Moore et al., 2021; Simkin, López-Calcagno & Raines, 2019). Plant biomass and biological yield are primarily determined by photosynthesis (Andralojc et al., 2018). The process of photosynthesis includes two stages of light reaction and dark reactions, whereas enzymes play an important role in the dark reaction (Yan, Yan & Gu, 2008).

Photosynthesis is essentially a series of enzymatic reactions that involves the *Ribulose 1,5-bisphosphate carboxylase* gene (*Rubisco*), *phosphoenolpyruvate carboxylase* gene (*PEP*), *ribose-5-phosphate isomerase* gene (*RPI*), *ribulose-phosphate 3-epimerase* gene (*RPE*), *malate dehydrogenase* gene (*MDH*), *fructose-1,6-bisphosphatase* gene (*FBP*), *Rubisco methyltransferase* gene (*RBCMT*), and *triosephosphate isomerase* gene (*TIM*) (Ciou et al., 2015; Liang et al., 2011; Whitney & Andrews, 2001; Wang et al., 2011). One major way of improving photosynthesis efficiency is to enhance the catalytic efficiency of enzymes related to photosynthesis. Although Rubisco catalyzes the fixation of CO_2_ in photosynthesis, a crucial step in the process of carbon fixation (Evans, 1986; Makino, Mae & Ohira, 1988), its catalytic efficiency is low, and in order to compensate for the low catalytic efficiency of Rubisco, plants accumulate Rubisco in large quantities in cells, making the content of Rubisco account for 20%–50% of soluble protein in plant cells (Sharwood, 2017; Galmés et al., 2019). Studies showed that limiting carboxylation and regeneration of ribulose 1, 5-bisphosphate (RUBP) could reduce the content and activity of ribulose 1, 5-bisphosphate carboxylation/oxygenase (Rubisco), down-regulate the expression levels of Rubisco proteins RBCL and RBCS, and thus reduced the photosynthetic rate of plants, and caused the imbalance of material and energy metabolism in plants (Dizengremel, 2001; Pellegrini et al., 2017). Rubsico activase (RCA) was responsible for the activation of Rubisco in leaves, and Rubisco could show its carboxylation/oxygenation activity only after activation, the activity of Rubisco in plants was therefore dependent on the activation of RCA (Perdomo, Buchner & Carmo-Silva, 2021). In addition, other genes related to photosynthesis were less reported.

*Eucalyptus camaldulensis* is one of the most widely planted tree species worldwide due to its rapid-growing, high-yield, and cold-resistant properties. In China, it has been widely planted in the southern region and has been used for a wide range of purposes (Luo et al., 2014). The bark of *E. camaldulensis* appears smooth with light color and gray mottling, as well as the branches, which are soft and drooping. It is suitable for street plantation as well as landscaping and is widely used as a shade and shelter tree (Hirsch et al., 2020). Recent studies have been conducted on improving the photosynthetic capacity of eucalyptus in order to improve afforestation quality and generate greater economic, ecological, and social benefits (Yang et al., 2018). Plant leaves are the primary sites for photosynthesis, transpiration, and respiration, photosynthesis, which affects the growth, development, and morphogenesis of plant (Salgado-Luarte & Gianoli, 2011; Liu et al., 2018). Although genetic manipulation can significantly enhance photosynthetic efficiency or productivity, there have been very few studies focused on eucalyptus photosynthetic genes. In order to study photosynthesis and related key genes in *E. camaldulensis* leaves during growth, photosynthetic indexes and transcriptome of young and mature leaves were analyzed, and key candidate genes were analyzed using bioinformatics. A deeper understanding of the mechanism of photosynthesis as well as improvements to the photosynthetic performance of *E. camaldulensis* will provide a valuable theoretical basis and scientific guidance for selective breeding and genetic engineering.

## Materials & Methods

### Plant material

In early April 2020, leaf samples of *E. camaldulensis* were collected from Southern China Experiment Nursery in Zhanjiang, Guangdong, China (21°15′30.69″N, 110°06′41.95″E). The young leaves (1-month-old) and mature leaves (4-month-old) of three healthy *E. camaldulensis* plants in the same direction and same height of second canopy were collected from the same half-sib families located on the Morehead R. Queensland (15°15′S, 143°34′E) with three biological replicates. The samples were kept in liquid nitrogen for RNA extraction.

### Determination of photosynthetic indexes

To measure the photosynthetic indexes of *E. camaldulensis* leaves, a portable photosynthetic apparatus (LI-6400XT) was used under natural light (from 10:00 to 12:00), which included the net photosynthetic rate (Pn), cond (Gs), trmmol (Tr), and leaf water use efficiency (WUE) = Pn/Tr. The stability of the environment was maintained by setting the light intensity at 1200 µmol m^−2^ s^−1^, the flow rate at 500 µmol s^−1^, and the CO_2_ concentration at the current atmospheric CO_2_ concentration ((400 ± 20) µmol mol^−1^), and the leaf temperature at 25 ± 1 °C. A total of three branches were selected, and three young and mature leaves were measured respectively in different parts of each branch with three biological replicates.

### Measurement of the light response curve

LI-6400XT portable photosynthetic apparatus was used to measure the light response curve of *E. camaldulensis* leaves from 10:00 to 16:00. The experimental apparatus was set as an open-air circuit keeping CO_2_ at 400 µmol mol^−1^, and the gas flow rate was 500 µmol s^−1^, and other parameters were set as default. A total of 17 photosynthetically active radiation (PAR, µmol m^−2^ s^−1^) were set using LED red and blue light sources, which included 2000, 1800, 1600, 1400, 1200, 1000, 800, 600, 400, 200, 150, 100, 80, 60, 40, 20 and 0 µmol m^−2^ s^−1^. The net photosynthetic rates of leaves were measured at different light intensities. Before determining the photosynthetic rate, they were induced for 2 min under the light intensity of 1200 µmol m^−2^ s^−1^, and the waiting time for each light intensity point was 120–200 s. A rectangular hyperbola model was used to draw the light response curve (Baly, 1935).

\begin{eqnarray*}{A}_{\mathrm{n}}(I)=aI{A}_{\mathrm{max}}/(aI+{A}_{\mathrm{max}})-{R}_{\mathrm{d}}. \end{eqnarray*}

*A*_n_(*I*) represents the net photosynthetic rate, *I* represents the light intensity, *a* represents the initial slope of the light response curve, *A*_max_ represents the maximum net photosynthetic rate, *R*_d_ represents the dark respiration rate. The analysis methods of one-way ANOVA and Duncan’s were used in SPSS19.0.

### RNA extraction and transcriptome sequencing

Total RNA was extracted using FastPure Universal Plant Total RNA Isolation Kit (Vazyme, Nanjing, China). The quantification of RNA was performed using Nanodrop™ 1000 (Thermo Fisher Scientific, Waltham, MA, USA). Agilent 2100 Bioanalyzer was used to measure the 28S/18S and RIN values. The quality and integrity of the RNA samples were estimated using agarose gel electrophoresis, and samples were resolved on 1% agarose gel. The enriched mRNA was reversely transcribed to form double-stranded cDNA, and the double ends of cDNA were repaired, and the adaptor was added for PCR amplification to construct the on-machine library. RNA libraries were constructed using the Illumina HiSeqTM 4000 platform at Gidio company. The statistical power of this experimental design, calculated in RNAseqPS is 0.446 (Guo et al., 2014).

### Sequencing data processing and analysis

TopHat (v2.0.10) was used to compare the reads with *E. grandis* reference genome (NCBI gcf_000612305.1). The assembly of sequences was performed by Cufflinks using reference annotation-based transcripts (RABT). The obtained transcript sequences were compared using BLAST with SwissProt, Gene Ontology, Cluster of Orthologous Groups of proteins, and Kyoto Encyclopedia of Genes and Genomes databases to annotate the function of the transcript.

The differentially expressed proteins were mapped to each term of the GO databases, and calculated the number of proteins in each term, and obtained the list of proteins with a certain GO function and the number of proteins. Hypergeometric tests were used to identify GO entries that were significantly enriched in differentially expressed proteins compared with the whole background protein, and the *p*-value of this hypothesis test could be calculated as follows:

\begin{eqnarray*}P=1-\sum _{i=0}^{m-1} \frac{ \left( \begin{array}{@{}c@{}} \displaystyle M\\ \displaystyle i\\ \displaystyle \end{array} \right) \left( \begin{array}{@{}c@{}} \displaystyle N-M\\ \displaystyle n-i\\ \displaystyle \end{array} \right) }{ \left( \begin{array}{@{}c@{}} \displaystyle N\\ \displaystyle n\\ \displaystyle \end{array} \right) } . \end{eqnarray*}

N was the number of proteins with GO annotation among all background proteins; n was the number of differentially expressed proteins in N; M was the number of proteins annotated as a specific GO term among all background proteins; m is the number of differentially expressed proteins annotated as a specific GO term. After the calculated *p*-value was corrected by Benjamin&Hochberg(BH method), the threshold was taken as correct-p-value ≤0.05. The major biological functions of differentially expressed proteins could be determined by GO functional significance enrichment analysis.

Pathway significant enrichment analysis took KEGG pathway as the unit, and hypergeometric test was used to find out the pathways that were significantly enriched in differentially expressed proteins compared with background proteins. The *p*-value of this hypothesis test could be calculated as follows:

\begin{eqnarray*}P=1-\sum _{i=0}^{m-1} \frac{ \left( \begin{array}{@{}c@{}} \displaystyle M\\ \displaystyle i\\ \displaystyle \end{array} \right) \left( \begin{array}{@{}c@{}} \displaystyle N-M\\ \displaystyle n-i\\ \displaystyle \end{array} \right) }{ \left( \begin{array}{@{}c@{}} \displaystyle N\\ \displaystyle n\\ \displaystyle \end{array} \right) } . \end{eqnarray*}

N was the number of proteins with pathway annotation among all background proteins; n was the number of differentially expressed proteins in N; M was the number of proteins annotated as a specific pathway among all background proteins; m was the number of differentially expressed proteins annotated as a specific pathway. After correction for multiple tests, pathways with *q*-value ≤0.05 were defined as those significantly enriched in differentially expressed proteins. Here, *q*-value is the *p*-value after FDR correction. The significant enrichment of pathway can identify the main biochemical metabolic pathways and signal transduction pathways involved in differentially expressed proteins.

Cuffdiff software (Trapnell et al., 2012) and EdgeR (Robinson, McCarthy & Smyth, 2010) were used to analyze the transcriptional expression and differences between mRNA sample pairs, respectively. DEGs were selected with a *p*-value <0.05 and —log2 fold change (log2FC)—>1 for subsequent analyses.

### Gene analysis

DEGs related to photosynthesis were screened out based on the data of and transcriptome. To analyze the relative molecular weight, theoretical isoelectric point, instability coefficient, hydrophilicity index as well as lipid solubility index of the gene, the ProtParam in ExPASy (http://web.expasy.org/protparam/) were employed. The secondary and tertiary structures of protein were analyzed using the SOPMA (https://npsa-prabi.ibcp.fr/cgi-bin/npsa_automat.pl?page=/NPSA/npsa_sopma.html) and SWISS-MODEL (https://swissmodel.expasy.org/interactive#seque-nce), respectively.

### RNA reverse transcription and QRT-PCR

M-MLV reverse transcriptase was adopted for synthesizing 2 µg RNA in line with Evo M-MLV RT Kit instructions (Accurate Biotechnology, China), and followed by a 1:10 dilution for subsequent experiments. The photosynthesis-related genes of *E. camaldulensis* were identified as verification genes. The total qRT-PCR reaction comprised of 20 µL containing 2 ×SYBR Green Pro Taq HS Premix (Tli RNaseH Plus) 10 µL, ROX Reference Dye 0.4 µL, forward primer (10 µmol L^−1^) 0.8 µL, reverse primer (10 µmol L^−1^) 0.8 µL, cDNA 2 µL, and RNase Free ddH_2_O 6 µL. The thermal profile was comprised of two segments: 30-S at 95 °C; 5-S denaturation at 95 °C, and 30-S annealing at 60 °C for altogether 40 cycles. Each reaction was repeated three times, and the *actin* gene was selected as a reference. Primer Express 2.0 Software (PE Applied Biosystems, USA) was applied in primer designing with default parameters. Table S1 displays the sequences of all the primers. The 2^−ΔΔCt^ method (Livak & Schmittgen, 2001) and Microsoft Excel software were used to analyze the obtained data.

## Results

### Photosynthesis analysis of the mature and young leaves in *E. camaldulensis*

**Figure 1:**
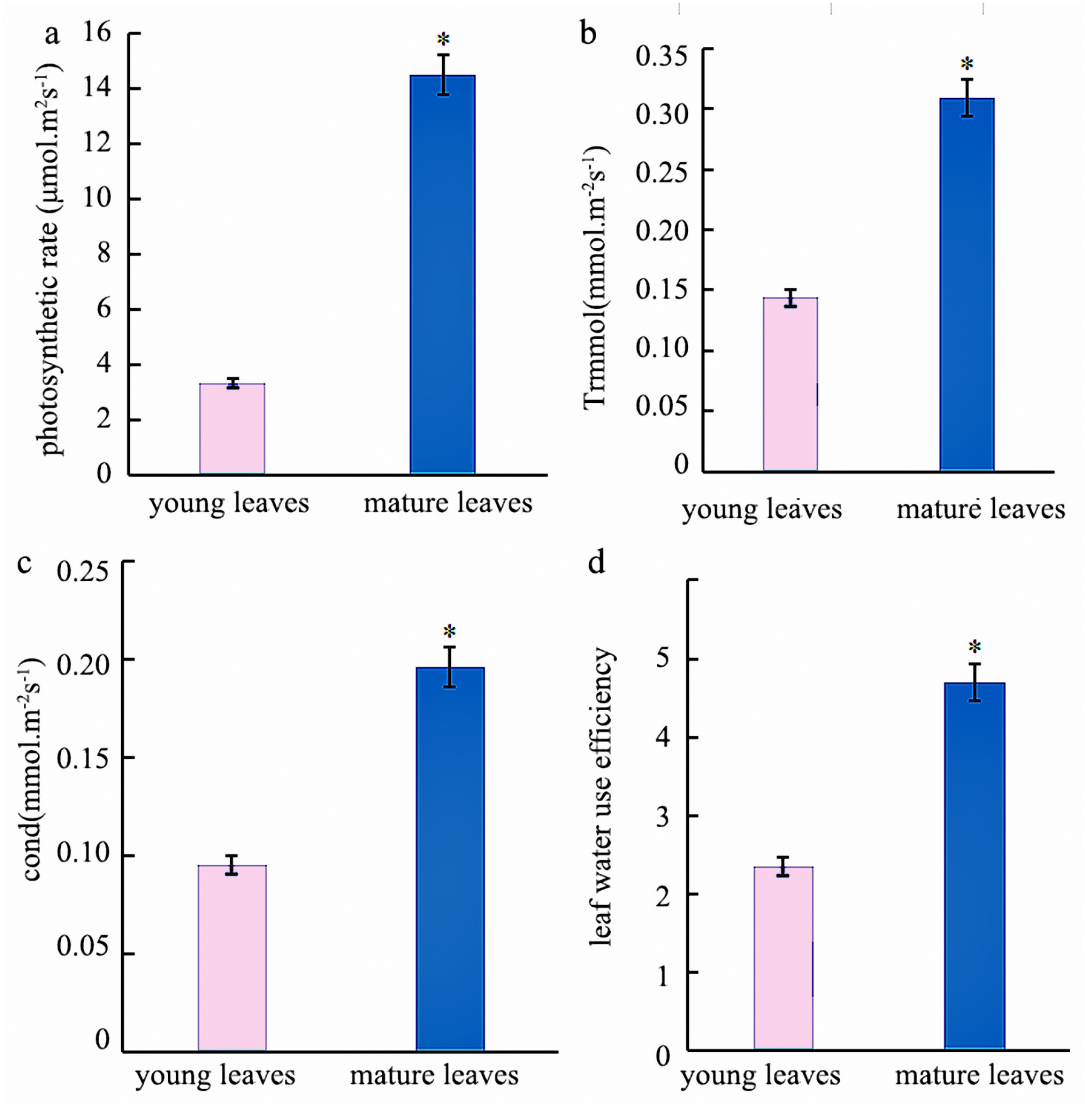
Analysis of the photosynthetic Indexes in young and mature leaves of *Eucalyptus camaldulensis*. (A) photosynthetic rate, (B) trmmol, (C) cond, (D) leaf water use efficiency. The asterisk indicated significantly different at *p* < 0.05 in the young and mature leaves of *E. camaldulensis*. Vertical bars indicate the standard error.

**Figure 2:**
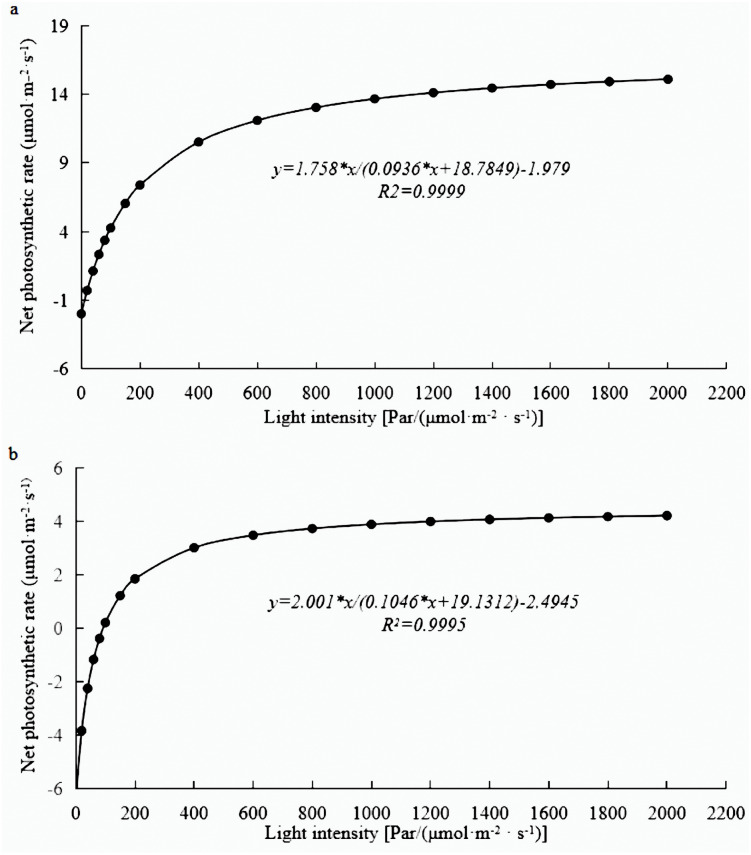
Light response curve of *E. camaldulensis*. (A) young leaves, (B) mature leaves.

Photosynthesis analysis revealed that mature leaves’ photosynthetic rate, trmmol, cond, and leaf water use efficiency were significantly higher than those of young leaves at a light intensity of 1200 µmol m^−2^ s^−1^ (Fig. 1). A photosynthesis rate is also called as a photosynthetic intensity, which is a measure of the intensity of photosynthesis. The photosynthetic rate can be expressed as carbon dioxide absorbed or oxygen released per unit time and per unit leaf area. The light response curve reflects the variation of plant photosynthetic rate with increasing light intensity; the net photosynthetic rate increased rapidly from 0 to 600 µmol m^−2^ s^−1^. However, the net photosynthetic rate decreased slower at light intensities of 600–1600 µmol m^−2^ s^−1^. A higher net photosynthetic rate was observed for mature leaves than for young leaves (Fig. 2).

### Differentially expressed genes related to photosynthesis in mature and young leaves of *E. camaldulensis*

We carried out a transcriptomic analysis on both young and mature leaf samples of *E. camaldulensis* to explore potential target genes. The overall raw/clean reads within each sample ranged between 6,914,804,063 and 8,686,921,500. This work also aligned sequence reads into the reference genome of *E. grandis*, and results indicated that >81% were map-able. The >92% Q30% and 48% GC concentrations suggested high-quality transcriptomic results for the subsequent analyses (Table 1).

**Table 1:** RNA sequencing data and quality control of *E. camaldulensis* leaves.

Sample	Raw date (bp)	Clean date (bp)	Mapped reads (%)	Q30 (%)	GC content (%)
Young leaves1	8667736800	8605418020	83.79	92.35	48.13
Young leaves2	7998500400	7945293590	83.05	92.43	48.67
Young leaves3	6958889600	6914804063	91.11	92.22	48.36
Mature leaves1	8686921500	8634113369	83.38	92.32	48.67
Mature leaves2	7575328200	7527501504	82.66	92.43	48.55
Mature leaves3	8577291000	8517745455	81.41	92.18	48.28

**Figure 3:**
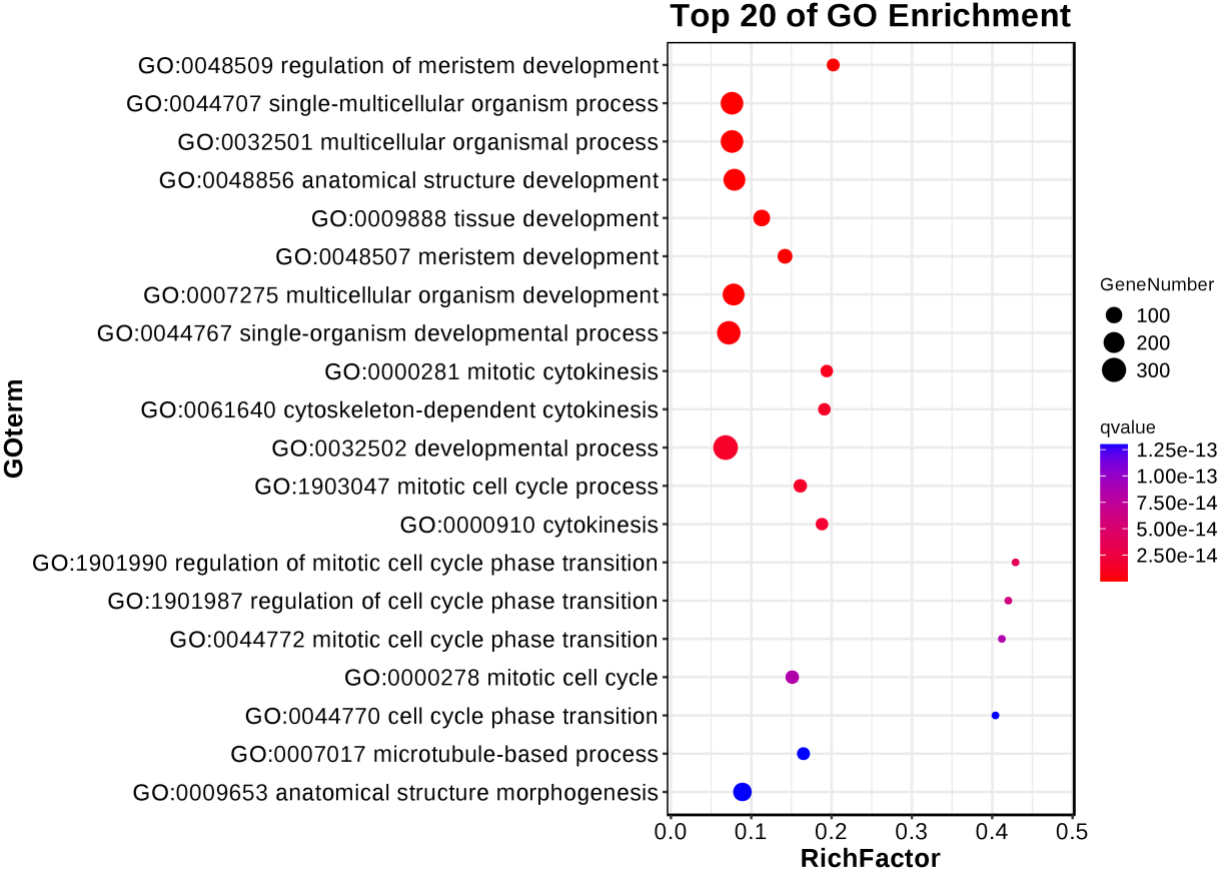
GO enrichment of differentially expressed genes in young and mature leaves of *E. camaldulensis*.

**Figure 4:**
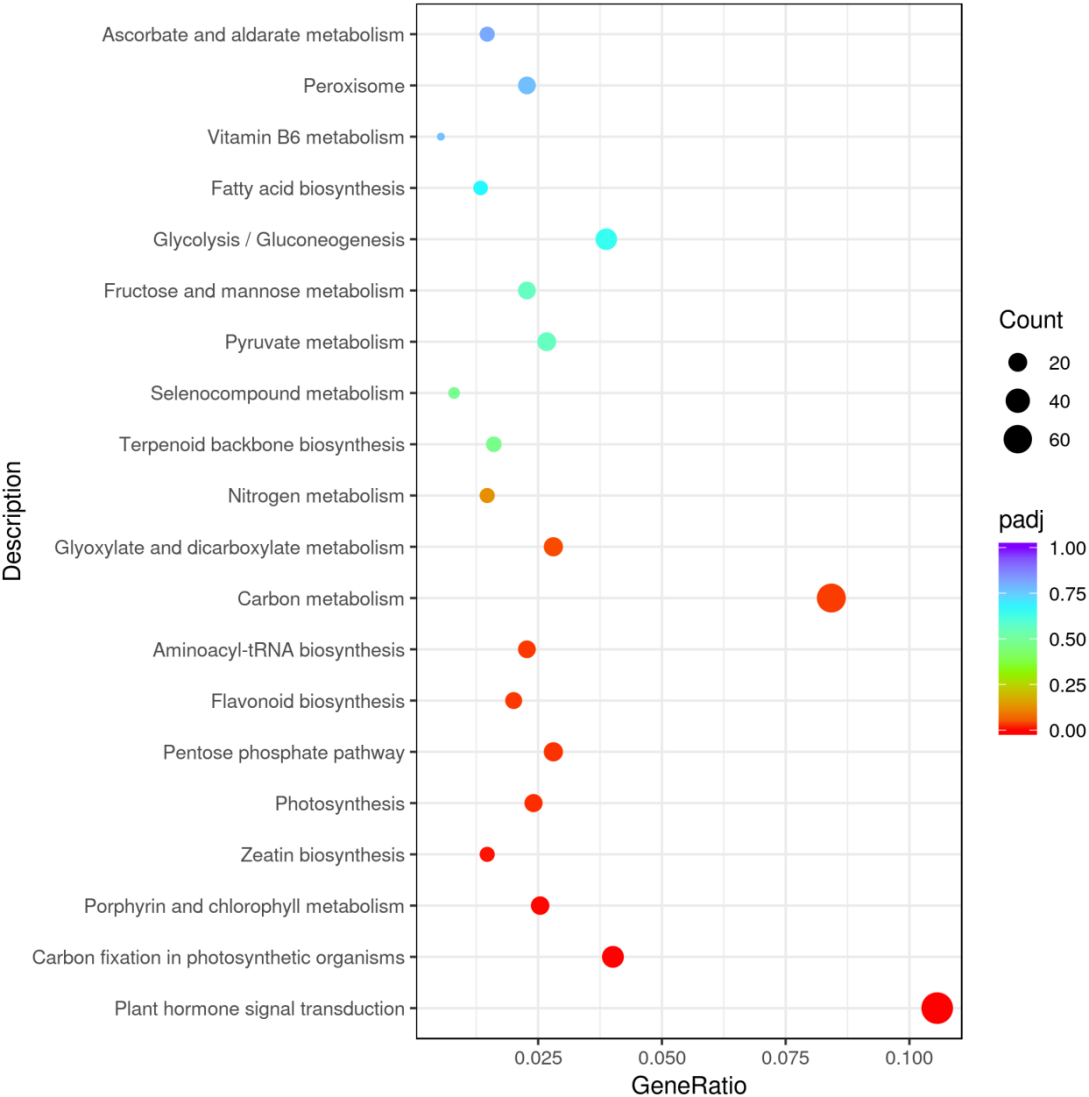
KEGG enrichment of differentially expressed genes in young and mature leaves of *E. camaldulensis*.

The results underlined that there were 18,443 differentially expressed genes (DEGs) in young and mature leaves in *E. camaldulensis*. A GO enrichment analysis indicated that DEGs were mainly enriched in the following processes: single-multicellular organism process, multicellular organism process, anatomical structure development, single-organism developmental process, multicellular organism development, and developmental process (Fig. 3). Furthermore, KEGG enrichment indicated that DEGs were mainly enriched in plant hormone signal transduction, carbon metabolism, photosynthesis, and carbon fixation in photosynthetic organisms (Fig. 4). We focused on the DEGs relating to photosynthesis, as mature leaves have a higher net photosynthetic rate than young leaves (Figs. 1 and 2).

A total of 18 important DEGs were identified in *E. camaldulensis* related to photosynthesis at different periods. Among ten *ribose-5-phosphate isomerases* genes (*RPI*), six were up-regulated in the mature leaves compared to young leaves, while the other four were down-regulated. There were four differentially expressed *rubisco methyltransferase* (*RBCMT*); two of them were up-regulated as well as two were down-regulated in the mature leaves compared to the young leaves. Furthermore, two *ribulose-phosphate-3-epimerase* genes (*RPE*) were found, and both of these were up-regulated in the mature leaves in comparison to the young leaves. On the other hand, two *triosephosphate isomerase* genes (*TIM*) were down-regulated in the mature leaves relative to the young leaves (Table 2).

**Table 2:** List of the differentially expressed genes in photosynthetic dark reactions of *E. camaldulensis*.

Gene number	Genes name	Log2 FC	FDR	Expression pattern (in mature leaves)
Unigene0035694	RPI1	1.819110678	6.22E−14	up
Unigene0091294	RPI2	5.15630553	8.70E−05	up
Unigene0056101	RPI3	4.517187259	8.70E−05	up
Unigene0058341	RPI4	2.04588154	2.89E−11	up
Unigene0039604	RPI5	2.186362763	9.61E−10	up
Unigene0088233	RPI6	1.680426069	4.85E−08	up
Unigene0098082	RPI7	−2.463303114	5.37E−09	down
Unigene0058337	RPI8	−2.375075931	6.34E−15	down
Unigene0078762	RPI9	−5.254852333	3.11E−11	down
Unigene0097988	RPI10	−1.426606232	4.13E−12	down
Unigene0032054	RBCMT1	1.918646352	2.30E−06	up
Unigene0103513	RBCMT2	3.248247344	1.80E−14	up
Unigene0068133	RBCMT3	−1.407208552	4.39E−15	down
Unigene0059727	RBCMT4	−1.367007493	1.77E−15	down
Unigene0029842	RPE1	1.609084978	3.56E−11	up
Unigene0028941	RPE2	1.908378016	1.37E−05	up
Unigene0058996	TIM1	−1.675416783	2.94E−15	down
Unigene0054879	TIM2	−1.552510135	8.12E−07	down

### Gene analysis bioinformatics analysis of genes related to photosynthesis

In order to further analyze the 18 DEGs related to photosynthesis in *E. camaldulensis*, bioinformatics methods were used to analyze them. Among the *RPI* genes of *E. camaldulensis, RPI2* was the longest amino acid encoded protein with a maximum relative molecular mass of 29,632.15 D, whereas the minimum relative molecular mass was *RPI9* (3852.50 D). *RPI2*, *RPI3*, and *RPI5* genes encoded for basic amino acids, whereas others encoded for acidic amino acids. A minimum instability index of 0.46 was observed in the case of *RPI3*. According to the hydrophilic index, the proteins encoded by *RPI1*, *RPI5*, *RPI8*, and *RPI9* genes were hydrophobic in nature, whereas others were hydrophilic proteins. The aliphatic index of *RPI* was found between 83.33 and 108.11. In the *RBCMT* genes of *E. camaldulensis*, *RBCMT2* was the longest amino acid encoded protein with a maximum relative molecular mass of 54957.68 D, while the minimum relative molecular mass was *RBCMT4* (9489.89 D). While *RBCMT1* encoded basic amino acids, others encoded acidic amino acids. The minimum instability index was 46.48 (*RBCMT3*). According to the hydrophilic index, the proteins encoded by *RBCMT* genes were hydrophobic in nature. The aliphatic index of *RBCMT* was found between 86.75 and 114.10. *RPE1* and *RPE2* encoded for the basic and acidic amino acids, respectively. The protein encoded by the *RPE1* gene was hydrophobic in nature, whereas the *RPE2* gene encoded for hydrophilic protein. *TIM1* and *TIM2* encoded acidic amino acids as well as hydrophilic proteins (Table 3).

**Table 3:** Basic information of genes involved in the dark reaction of photosynthesis in *E. camaldulensis*.

Gene number	Gene name	aa length	Relative molecular mass/D	PI	Instability index	Hydrophilic index	Aliphatic index
Unigene0035694	RPI1	159	16908.35	5.00	38.01	0.07	101.82
Unigene0091294	RPI2	278	29632.15	7.91	26.69	−0.06	102.09
Unigene0056101	RPI3	63	7320.58	8.07	43.51	−0.27	103.49
Unigene0058341	RPI4	42	4594.17	4.65	10.68	−0.34	83.33
Unigene0039604	RPI5	143	15633.40	7.87	30.18	0.21	103.50
Unigene0088233	RPI6	44	4742.45	4.33	36.14	−0.03	84.09
Unigene0098082	RPI7	60	6452.19	4.01	22.79	−0.14	81.17
Unigene0058337	RPI8	243	25772.65	5.12	19.46	0.05	105.10
Unigene0078762	RPI9	37	3852.50	6.81	0.46	0.23	108.11
Unigene0097988	RPI10	120	12493.08	5.14	44.22	−0.08	96.00
Unigene0032054	RBCMT1	126	13465.66	10.27	62.53	−0.15	86.75
Unigene0103513	RBCMT2	488	54957.68	5.06	66.53	−0.22	98.91
Unigene0068133	RBCMT3	311	35655.71	4.93	46.48	−0.25	96.88
Unigene0059727	RBCMT4	82	9489.89	5.06	73.10	−0.06	114.10
Unigene0029842	RPE1	277	29516.20	8.86	46.52	0.17	106.53
Unigene0028941	RPE2	172	19115.26	5.54	37.47	−0.11	84.94
Unigene0058996	TIM1	246	26350.13	5.21	27.54	0.02	102.15
Unigene0054879	TIM2	52	5284.97	5.95	12.64	0.07	90.00

**Figure 5:**
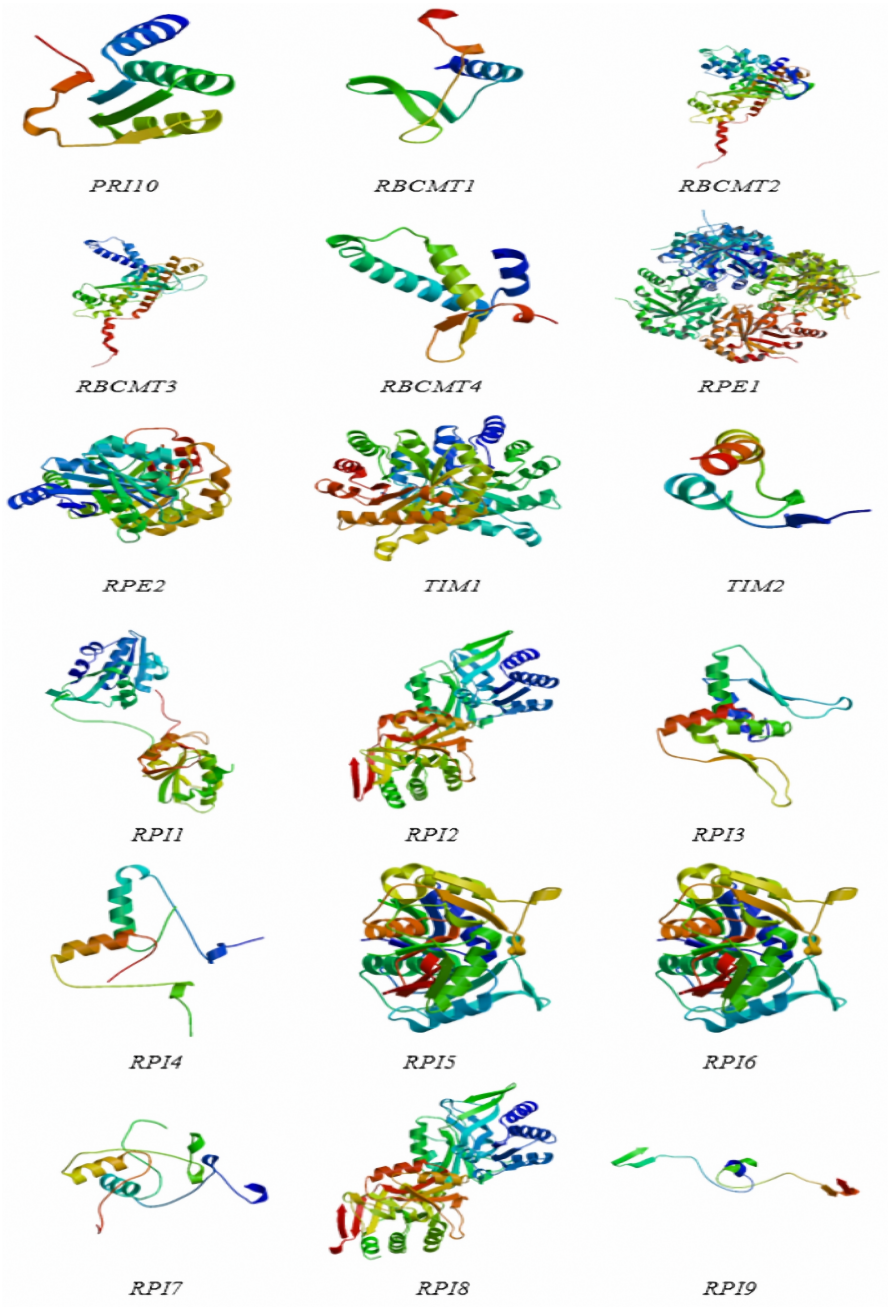
The protein structures of dark reaction proteins of photosynthesis in *E. camaldulensis*.

The protein structures of the *RPI*, *RBCMT*, *RPE*, and *TIM* gene consisted of α-helix, extension chain, random coil, and β-angle structure (Table 4 and Fig. 5). These results showed that the proportion of RPI protein α-helix structures was usually higher than others, with the lowest is β-angle structure. Similarly, the proportion of RBCMT protein α-helix and random coil structure was also usually higher than that of protein with other structures. The highest proportion of α-helix, as well as the lowest ratio of β-angle, was found in the secondary structure of RPE and TIM proteins.

**Table 4:** Analysis of the secondary structure of dark reaction proteins of photosynthesis in *E. camaldulensis*.

Protein	α-helix		Extending chain		Random coil		β-angle	
	aa length	Proportion %	aa length	Proportion %	aa length	Proportion%	aa length	Proportion %
RPI1	67	42.14	33	20.75	49	30.82	10	6.29
RPI2	104	37.41	59	21.22	89	32.01	26	9.35
RPI3	29	46.03	12	19.05	15	23.81	7	11.11
RPI4	15	35.71	9	21.43	12	28.57	6	14.29
RPI5	51	35.66	33	23.08	41	28.67	18	12.59
RPI6	17	38.64	9	20.45	14	31.82	4	9.09
RPI7	17	28.33	17	28.33	19	31.67	7	11.67
RPI8	100	41.15	50	20.58	68	27.98	25	10.29
RPI9	15	40.54	9	24.32	5	13.51	8	21.62
RPI10	40	33.33	27	22.50	43	35.83	10	8.33
RBCMT1	32	25.40	30	23.81	55	43.65	9	9.17
RBCMT2	212	43.44	62	12.70	194	39.75	20	4.10
RBCMT3	164	52.73	39	12.54	96	30.87	12	3.86
RBCMT4	54	65.84	5	6.10	22	26.83	1	1.22
RPE1	129	46.57	48	17.33	77	27.80	23	8.30
RPE2	76	44.19	30	17.44	47	27.33	19	11.05
TIM1	119	48.37	37	15.04	72	29.27	18	7.32
TIM2	18	34.62	9	17.31	17	32.69	8	15.38

### Q RT-PCR analysis of key genes related to photosynthesis

**Figure 6:**
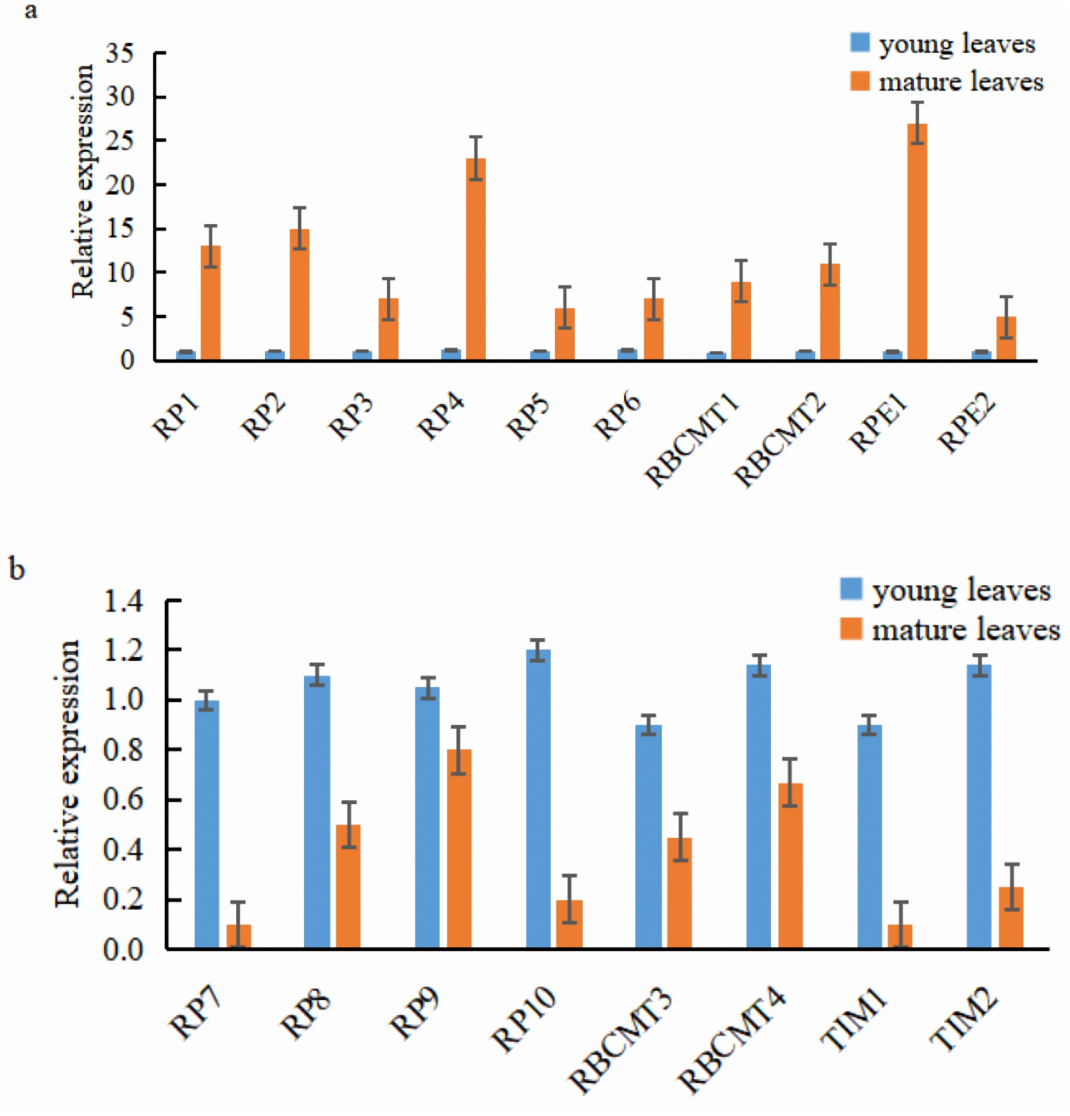
Expression of the target genes in young and mature leaves of *E. camaldulensis* as determined by the quantitative real-time PCR. (A) genes were up-regulated in the mature leaves, (B) genes were down-regulated in the mature leaves.

Ten of the 18 target genes selected for analysis were over-expressed in mature leaves, namely *RPI1*, *RPI2*, *RPI3*, *RPI4*, *RPI5*, *RPI6*, *RBCMT1*, *RBCMT2*, *RPE1*, and *RPE2*. The higher expression of these genes in mature leaves than younger ones was verified by qRT-PCR (Fig. 6A). On the other hand, eight target genes were down-regulated in the mature leaves viz., *RPI7*, *RPI8*, *RPI9*, *RPI10*, *RBCMT3*, *RBCMT4*, *TIM1* as well as *TIM2* and the qRT-PCR verified that their higher expression in young leaves than mature leaves (Fig. 6B). A consistent pattern of results was found between the results of the qRT-PCR and the RNA sequencing.

## Discussion

### Compare photosynthesis between young and mature leaves in *E. camaldulensis*

The rate of photosynthesis is closely related to the leaf age, and it changes with the age of the leaves. The photosynthetic rate showed a single peak curve from leaf initiation to senescence and wilting (Smith et al., 1997). In this study, the photosynthetic rate and trmmol of mature leaves were found to be higher than those of young leaves in *E. camaldulensis*. Similar findings were observed in Chinese fir, where the light absorption capacity and transformation capacity of old leaves were reported to be higher than those of young leaves (Li et al., 2017). This phenomenon may be possible due to the higher vulnerability of chlorophyll in young leaves to intense light. An increase in the net photosynthetic rate leads to higher stomatal conductance followed by more CO_2_ into carboxylated parts of the plant cells for photosynthesis. The increase of stomatal conductance also accelerated the loss of water, leading to the continuous increase of transpiration rate (De Souza et al., 2020). The light response curve reflects the change in net photosynthetic rate with the change of light intensity (Poorter & Navas, 2003), which provides the estimate of photosynthetic rate, light saturation point, light compensation point as well as other important ecological and physiological parameters of plants (Cannell & Thornley, 1998). Therefore, in this study, we analyze the light response curves of young and mature leaves of *E. camaldulensis* to understand the photosynthetic efficiency and growth of eucalyptus.

### Key genes associated with photosynthesis in *E. camaldulensis*

The high-throughput sequencing provided huge data with high quality, which was suitable for the transcriptome study of *Eucalyptus* species, and improved our understanding about the molecular mechanisms in *Eucalyptus* (Morozova & Marra, 2008; Kadam et al., 2013; Xiao et al., 2020). Although, the role of Rubisco and PEP (two key enzymes in photosynthesis dark reactions) have been widely explored, there is limited information available on *ribose-5-phosphate isomerase* (*RPI*), *rubisco methyltransferase* (*RBCMT*), *ribulose-phosphate 3-epimerase* (*RPE*), and *triosephosphate isomerase* (*TIM*) genes. Enzymes play an important role in the dark reaction of photosynthesis; therefore, *RPI*, *RBCMT*, *RPE*, and *TIM* genes were selected as the research objects.

RPI is a highly conserved protease that is ubiquitous in many organisms and plays a central role in the pentose phosphate pathway (PPP). It is involved in the reversible isomerization of ribose-5-phosphate (R5P) and ribulose 5-phosphate (Ru5P) between prokaryotes and eukaryotes as well as in the Calvin cycle of carbon dioxide fixation in plants (Wamelink et al., 2010; Gontero, Cárdenas & Ricard, 1988). According to the reports, RPI is an essential enzyme in the Calvin cycle in peas and spinach (Woodruff & Wolfenden, 1979; Park & Anderson, 1973). The photosynthetic rate of mature leaves was higher than that of young leaves in *E. camaldulensis*, genes such as *RPI1*, *RPI2*, *RPI3*, *RPI4*, *RPI5*, and *RPI6* were up-regulated in the mature leaves. Among the *RPI* genes of *E. camaldulensis, RPI2* was the longest amino acid encoded protein. *RPI2*, *RPI3*, and *RPI5* genes encoded for basic amino acids. The proteins encoded by *RPI1* and *RPI5* genes were hydrophobic in nature. The results indicate that the high expression of *RPI* genes in mature leaves could produce more PRI enzymes for photosynthesis.

Rubisco is a bifunctional enzyme and is widely available in the chloroplast matrix of plants. It can catalyze both the carboxylation reaction of C_3_ for photosynthesis and the oxygenation reaction of C_5_ for photorespiration. In photosynthesis, Rubisco was present at the intersection of carbon oxidation and carbon reduction, and RBCMT was the rubisco methyltransferase (Miyazawa et al., 2020). The expression of *RBCMT1* and *RBCMT2* was positively correlated with the net photosynthetic rate. *RBCMT2* was the longest amino acid encoded protein in *E. camaldulensis*. *RBCMT1* encoded basic amino acids. According to the hydrophilic index, the proteins encoded by *RBCMT* genes were hydrophobic in nature.

RPE can play a vital role in the development of the NADPH pool, and PPP, which can convert monosaccharides, such as glucose, into the nucleotide precursor of pentose sugars. Additionally, RPE can convert the ribulose-5-phosphate into xylulose 5-phosphate in the Calvin cycle (Lykins et al., 2018). The photosynthetic rate of mature leaves was higher than that of young leaves in *E. camaldulensis*. *RPE1* and *RPE2* were up-regulated in mature leaves, which indicates that these may be the key genes to enhancing photosynthesis. *RPE1* encoded for the basic amino acids, and *RPE2* encoded for the acidic amino acids. The protein encoded by the *RPE1* gene was hydrophobic in nature, whereas the *RPE2* gene encoded for hydrophilic protein. Furthermore, *RP7*, *RP8*, *RP9*, *RP10*, *RBCMT3*, *RBCMT4*, *TIM1*, and *TIM2* genes were highly expressed in young leaves of *E. camaldulensis*, indicating that they may be involved in the negative regulation (Katrin et al., 1994). These results provide a basic structure for understanding the *RPI*, *RBCMT*, *RPE*, and *TIM* genes and exploring the functions of genes for further research in *E. camaldulensis*. Through genetic transformation, enhancing photosynthetic efficiency or productivity by using these genes to ensure the better afforestation quality of eucalyptus plantations and to generate greater social, ecological, and economic value.

## Conclusions

The present study revealed the characteristics of photosynthesis and transcriptome of young and mature leaves in *E. camaldulensis*. A total of 18 target genes related to photosynthesis were analyzed using bioinformatics methods.The RPI genes were differentially expressed in mature leaves, six of which were up-regulated, while others were down-regulated. Among the four RBCMT genes, two were up-regulated. Mature leaves exhibited up-regulation in two RPE genes, while young leaves showed down-regulation in two TIM genes. The basic physical and chemical properties, including relative molecular weight, theoretical isoelectric point instability coefficient, hydrophilic index, and lipid solubility index, were systematically analyzed. The obtained protein structures consist of α-helix, extension chain, random coil, and β-angle structure. The outcome of this study provides a platform to understand the basic characteristics of *RPI*, *RBCMT*, *RPE*, and *TIM* genes, and further explore the functions of the genes in eucalyptus. Our findings provide profuse data resources for further research on the growth and development of *E. camaldulensis,* as well as the function and regulation of photosynthesis-related genes.

##  Supplemental Information

10.7717/peerj.14351/supp-1 Supplemental Information 1 List of primers for real-time quantitative PCR reactions

10.7717/peerj.14351/supp-2 Supplemental Information 2 Light response curves raw data

10.7717/peerj.14351/supp-3 Supplemental Information 3 Photosynthetic rate raw data

10.7717/peerj.14351/supp-4 Supplemental Information 4 Q RT-PCR raw data

10.7717/peerj.14351/supp-5 Supplemental Information 5 Young leaves were defined from the first unfolded leaf on the branch to the fifth leaf on the branch, and the fully expanded and healthy leaves on the branch the upper arrow; Mature leaves were defined as the 6th - 10th leaf on the branch from the first u

10.7717/peerj.14351/supp-6 Supplemental Information 6 18443 differentially expressed genes in young and mature leaves in *E. camaldulensis*, and the detailed information of the 18443 DEGs (such as Log2FC, padj, annotation information et al.)

10.7717/peerj.14351/supp-7 Supplemental Information 7 Go analysis

10.7717/peerj.14351/supp-8 Supplemental Information 8 KEGG analysis

## Additional Information and Declarations

### Data Availability

The following information was supplied regarding data availability: The RNA-Seq reads are available at Sequence Read Archive (SRA): PRJNA867010.
