# Supplementary material for: Comparative transcriptomics and bioinformatics analysis of genes related to photosynthesis in Eucalyptus camaldulensis
Source: PeerJ. 2022 Nov 11;10:e14351. doi: 10.7717/peerj.14351 (PMC9661968; doi:10.7717/peerj.14351)
Supplement: Supplemental Information 1 [file peerj-10-14351-s001.docx]

**Table S1.** List of primers for real-time quantitative PCR reactions

| Target gene | Primer sequence(5’-3’) |
| --- | --- |
| RPI1 | F:TCGCCAAGACCATCTACAGC |
| RPI1 | R:AATGGCCCCGAACCAGAGAAGG |
| RPI2 | F:GGCTGCTACCCTCCGTTATC |
| RPI2 | R:GCTGATGATGCGTTCGTGAC |
| RPI3 | F:GTGAGACCAGATGGGGAAGC |
| RPI3 | R:CGCACTCCAAGTAAGGGAGG |
| RPI4 | F:TGTATCGGCGTCTCCCCATT |
| RPI4 | R:CAGTTGGTGCCGAAGATGAG |
| RPI5 | F:ACTGCATTTGTATCGGCGTC |
| RPI5 | R:TTGGCGTGGTTGTAGTAGCC |
| RPI6 | F:GGTCTTTTGTTCCAAGGGGG |
| RPI6 | R:ACGCCGAGTGATTTACCAGG |
| RPI7 | F:CTGTGGTTATGGGGAACATGGAA |
| RPI7 | R:GTGGAGAGCCGAAGAAGAAGAGG |
| RPI8 | F: ACATCGTCGGGGTCCCGACCTC |
| RPI8 | R: ATCCGCCACCACCACGAACTTG |
| RPI10 | F: GGACGACCTCAAGCGGATTGC |
| RPI10 | R: AGGGCCTGCTGGTGGGTCTGG |
| RBCMT1 | F:CTCGGGCTACTTCATCGTCC |
| RBCMT1 | R:GGTACAGGTCGGCGTAGATG |
| RBCMT2 | F:GCCCCTTCATCCTCAACCTC |
| RBCMT2 | R:GCAAATCGTCGGAACAAGC |
| RBCMT3 | F: ACTTATTGCCAGCCTAAGAAGGAT |
| RBCMT3 | R: CCATCTAAGCCGAGTCTGTAATGT |
| RBCMT4 | F:AAGAGGAGCTGGCTGAGCTGCAA |
| RBCMT4 | R:ACACAAGGTTTTGGCCACGAAGTC |
| RPE1 | F:CGGCGATAAGCAGACCAAGA |
| RPE1 | R:ACAGATCAGAACACAGGCCG |
| RPE2 | F:TGCGTCGAAGGGGTTGAATAC |
| RPE2 | R:ATCATGGGCTGAAGGCTGCT |
| TIM1 | F: GTGTTGGGGAGACTCTTGAGCAG |
| TIM1 | R: TCGGGACCAGTATTTTCATGTAGC |
| TIM2 | F: GGAGGGTCTGTAAATGGAGGCAA |
| TIM2 | R: ATGTGTGCTGGTCACCCCTTATT |
| Eucalyptus Actin | F:AGATGACCCAGATTATGTTTGAGACCTTC |
| Eucalyptus Actin | R: ACCATCACCAGAATCCAACACAATACC |
