# Supplementary material for: Comparative transcriptomics and bioinformatics analysis of genes related to photosynthesis in Eucalyptus camaldulensis
Source: PeerJ. 2022 Nov 11;10:e14351. doi: 10.7717/peerj.14351 (PMC9661968; doi:10.7717/peerj.14351)
Supplement: Supplemental Information 5 [file peerj-10-14351-s005.pdf]

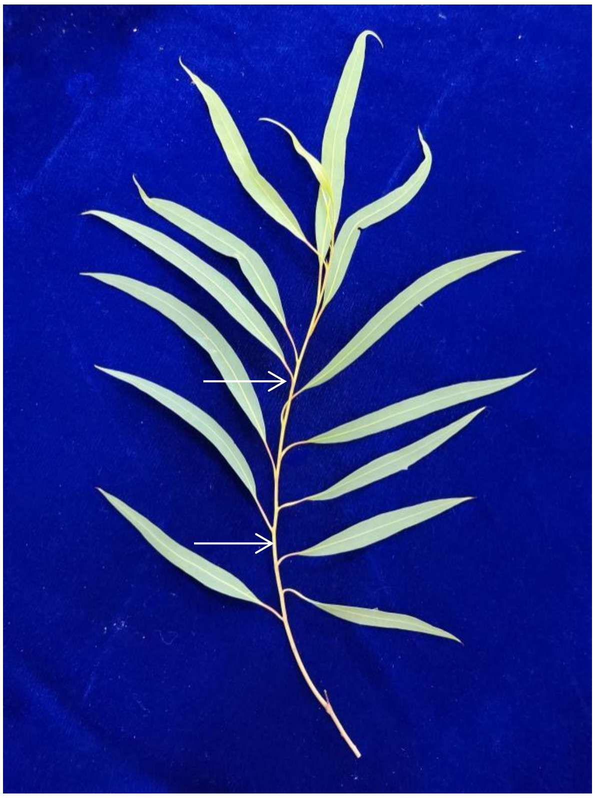

Young leaves were defined from the first unfolded leaf on the branch to the fifth leaf on the branch, and the fully expanded and healthy leaves on the branch the upper arrow; Mature leaves were defined as the 6th - 10th leaf on the branch from the first unfolded leaf on the branch, as well as the intact and healthy leaves on the branches between the two arrows.
